# Design and Testing of a Flexible Inclinometer Probe for Model Tests of Landslide Deep Displacement Measurement

**DOI:** 10.3390/s18010224

**Published:** 2018-01-14

**Authors:** Yongquan Zhang, Huiming Tang, Changdong Li, Guiying Lu, Yi Cai, Junrong Zhang, Fulin Tan

**Affiliations:** 1 Faculty of Engineering, China University of Geosciences, Wuhan 430074, China; zhangyq@cug.edu.cn (Y.Z.); caiyi2015@cug.edu.cn (Y.C.); zjr@cug.edu.cn (J.Z.); tanfulin@cug.edu.cn (F.T.); 2 School of Mechanical Engineering and Electronic Information, China University of Geosciences, Wuhan 430074, China; luguiying0510@163.com

**Keywords:** landslide model test, displacement monitoring, flexible inclinometer probe, accelerometer, three-spline interpolation

## Abstract

The physical model test of landslides is important for studying landslide structural damage, and parameter measurement is key in this process. To meet the measurement requirements for deep displacement in landslide physical models, an automatic flexible inclinometer probe with good coupling and large deformation capacity was designed. The flexible inclinometer probe consists of several gravity acceleration sensing units that are protected and positioned by silicon encapsulation, all the units are connected to a 485-comunication bus. By sensing the two-axis tilt angle, the direction and magnitude of the displacement for a measurement unit can be calculated, then the overall displacement is accumulated according to all units, integrated from bottom to top in turn. In the conversion from angle to displacement, two spline interpolation methods are introduced to correct and resample the data; one is to interpolate the displacement after conversion, and the other is to interpolate the angle before conversion; compared with the result read from checkered paper, the latter is proved to have a better effect, with an additional condition that the displacement curve move up half the length of the unit. The flexible inclinometer is verified with respect to its principle and arrangement by a laboratory physical model test, and the test results are highly consistent with the actual deformation of the landslide model.

## 1. Introduction

Landslides are common and widely reported all over the world. To effectively control landslides, scholars have conducted considerable experimental research on landslides [1,2,3,4,5]. Landslide physical model testing is a very important and effective means of research [6,7,8]. Landslide physical model testing has gone through qualitative, semi-quantitative and quantitative development stages. Today, more accurate quantitative analysis of landslides is needed. The rationality and accuracy of data acquisition in the testing process play decisive roles.

However, to facilitate experimentation in the laboratory, the proportions of the physical model must be narrowed according to similarity theory [9]; thus, the size of the physical model is usually small, leading to difficulty in the direct application of some conventional landslide monitoring instruments, such as GPS [10,11], InSAR [12,13], LiDAR [14,15] and inclinometers [16], to the model test. Therefore, it is of great value to conduct research on and develop parameter measurement instruments for model testing.

The existing instruments and methods for measuring displacement in a landslide physical model test, such as 3D laser scanning, close-range photography, and the displacement meter, primarily target surface displacement [4,17]. Therefore, few mature instruments are available for measuring the distribution of vertical displacement. However, fortunately, certain traditional deep displacement measuring instruments for landslides in the field can be used as important references.

The current referenced deep displacement measuring instruments include time-domain reflectometry (TDR), fibre Bragg grating and drilling inclinometers. The TDR measuring technique uses a coaxial cable that is buried in the landslide for coupling the deformation of the landslide and converting the local landslide deformation through reflection wave impedance analysis [18,19,20]. Fibre Bragg grating is a new method for measuring landslide displacement in which the instrument is buried in the landslide for mapping the deformation information through the modulation and analysis of optical signal transmission changes in the fibre Bragg grating circuits [21,22,23]. With similar measurement models and their advantages and disadvantages, both measuring methods are able to adapt to the requirement of real time automatic measurement, but the measurement results are greatly influenced by the embedding process. Furthermore, it is difficult to form a standard quantitative measurement based on the strain of the reference plate, the column, the pipe, and even the line to measure the conversion amount, and the conversion from strain to displacement is not determined. In principle, the cable or fibre Bragg grating must be tight when buried and is thus in danger of snapping during deformation, especially when the soil is soft. The deformation of the soil mass is likely to be influenced by the datum plate, pillar, pipe or line and thus does not reflect the actual deformation of the landslide. The borehole clinometer is a widely recognized measuring instrument and can react to the deformation in the deep part of the landslide. This instrument contains a single-point sliding inclinometer and a multi-point fixed inclinometer [24,25,26]. The single-point sliding inclinometer has just a single sensing unit that slides in the inclinometer pipe and records each data point at different moments. The multi-point fixed inclinometer contains several sensing units that are statically installed in the borehole, and it measures all of the data at the same time [27]. The former is inexpensive but requires manual manipulation, and later maintenance costs are high; the latter is costly but easy to automate.

Based on the principle of the inclinometers used for boreholes, in this project we developed a flexible inclinometer probe for a model test of deep landslide displacement measurement. Compared to the existing device, the flexible inclinometer probe has the following features: its flexible encapsulation structure makes it very well coupled with landslide deformation and enables it to withstand more serious local bending, while the z-axis position is more difficult to control. The main contents of this paper include the basic measuring principle for the deep displacement physical model test, the design of the flexible probe, the calibration and correction of measurement data, the installation method for the flexible probe in a landslide physical model test and a measurement case.

## 2. Measuring Principle

### 2.1. Angle Measurement Units with Gravity Component Accelerometer

In practical engineering, the displacement of a landslide is generally relative to the displacement of the geodetic coordinate system; therefore, to measure the position change caused by any sliding, one must locate a relatively fixed reference in the geodetic coordinate system as the benchmark. The geomagnetic and gravitational fields are two optional physical quantity references commonly used in measurements. Between them, the geomagnetic field is not an ideal reference because of its weak strength and vulnerability to experience impact from the surrounding environment. The unit of the gravitational field strength is gravitational acceleration, g, and it has a strong anti-interference and regional distribution stability that makes it an ideal reference for the measurement of attitude changes.

**Figure 1:**
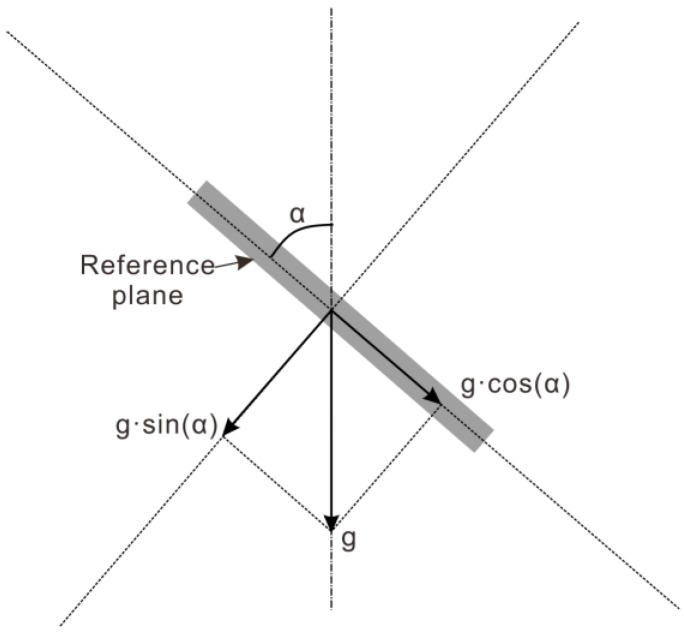
Principle of measuring an angle with an accelerometer.

As shown in Figure 1, based on the gravitational acceleration, g, a gravity component associated with the inclination angle α is generated on the sensitive axis of the accelerometer when the sensitive axis and g direction are non-vertical, and the inclination angle α can be calculated when the gravity component is measured [28,29,30]. What is especially illustrated is that only the inclination angle with respect to the direction of the plumb line can be measured, and horizontal rotation cannot be based on this principle.

The XYZ sensitive axis of the accelerometer in the initial state is defined to coincide with the coordinate system xyz axes. Then, the measured value of the three-axis acceleration can be expressed as

(1) $$A=\left[\begin{array}{c} A_{x}\\ A_{y}\\ A_{z} \end{array}\right]=C\left[\begin{array}{c} 0\\ 0\\ g \end{array}\right]+a,$$

where $A_{x}$, $A_{y}$ and $A_{z}$ are the accelerations on the xyz axes; C is the rotation matrix; ${\left[\begin{array}{ccc} 0 & 0 & g \end{array}\right]}^{T}$ is the gravity acceleration component of the three axes in the initial state; and a is the motion acceleration.

**Figure 2:**
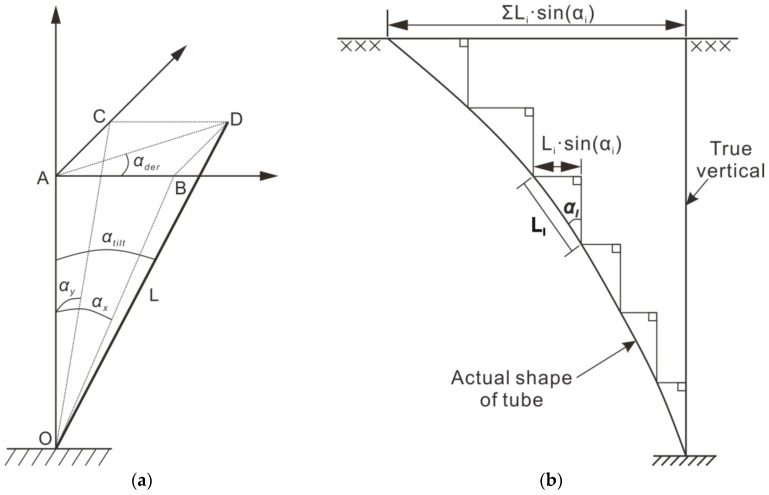
Calculation of displacement through the angle diagram: (**a**) Schematic diagram of angle to displacement conversion; (**b**) schematic diagram of cumulative displacement curves [31].

When the accelerometer is in constant motion or a static state, the line acceleration a ≅ 0. Compared with the gravitational acceleration, the line acceleration (a → 0) of the landslide is far less than g and is negligible in the monitoring. Meanwhile, the soil layer will not reverse in the progress of sliding, thus, the acceleration of the z-axis can be ignored. Then, Equation (1) can be more simply expressed as

(2) $$\left[\begin{array}{l} A_{x}\\ A_{y} \end{array}\right]=\left[\begin{array}{l} g\cdot \sin\left(\alpha_{x}\right)\\ g\cdot \sin\left(\alpha_{y}\right) \end{array}\right],$$

which is

(3) $$\left\{ \begin{array}{l} \alpha_{x}=\arcsin\left(\frac{A_{x}}{g}\right)\\ \alpha_{y}=\arcsin\left(\frac{A_{y}}{g}\right) \end{array}\right.,$$

where $\alpha_{x}$ and $\alpha_{y}$ are the inclination angles in the x-axis and y-axis directions, respectively, as shown in Figure 2.

Through the relationship shown in Figure 2, the integrated dip angle is

(4) $$\alpha_{tilt}=\arccos\left(\sqrt{1-\sin^{2}(\alpha_{x})-\sin^{2}(\alpha_{y})}\right),$$

By measuring in a right-handed sense about the +z-axis with zero azimuth on the +x-axis, the azimuth is

(5) $$\alpha_{azi}=\{\begin{array}{c} \arctan\left(\frac{A_{y}}{A_{x}}\right),\;\left(A_{x}>0\right)\\ \begin{array}{l} \arctan\left(\frac{A_{y}}{A_{x}}\right)+\pi ,\;\left(A_{x}<0,A_{y}>0\right)\\ \arctan\left(\frac{A_{y}}{A_{x}}\right)-\pi ,\;\left(A_{x}<0,A_{y}<0\right) \end{array} \end{array},$$

### 2.2. Measurement of the Distribution of Lateral Displacement by the Accelerometer Cluster

As shown in Figure 2a, the accelerometer cannot obtain the horizontal displacement of the landslide directly; rather, it measures the inclination angle and tendency of the carrier and obtains the projection of length L in the horizontal plane along the inclination and azimuth angle that is horizontal to the displacement of the landslide by assuming that the deflection angle of the carrier within a finite height range L can be approximated to the same value. Then, the horizontal displacement in length L is

(6) $$\left\{ \begin{array}{l} D_{x}=AB=L\cdot \sin\left(\alpha_{x}\right)\\ D_{y}=AC=L\cdot \sin\left(\alpha_{y}\right)\\ D=AP=L\cdot \sin\left(\alpha_{tilt}\right),\;\mathrm{or}\left(\sqrt{D_{x}^{2}+D_{y}^{2}}\right) \end{array}\right.,$$

where $D_{x}$ and $D_{y}$ are displacement along the x and y axes, respectively, and D is the overall displacement along the $\alpha_{tilt}$ direction.

As shown in Figure 2b, the landslide body is divided into n segments along the depth direction according to the interval L, and the axis consists of n units in series connection. It is clear that the shorter the separation distance L is, the closer it will be to the real situation. Assuming that L is short enough to reflect the local deformation of the landslide by n units, the displacement of the unit k location can be expressed as

(7) $$\left\{ \begin{array}{l} D_{x,k}=\sum_{i=1}^{k}L_{i}\cdot \sin\left(\alpha_{x,i}\right)\\ D_{y,k}=\sum_{i=1}^{k}L_{i}\cdot \sin(\alpha_{y,i})\\ D_{k}=\sqrt{D_{x,k}^{2}+D_{y,k}^{2}} \end{array}\right.,\;k=1,2,3\ldots ,n,\;i=1,2,3,\ldots ,k,$$

where i is the number of each unit under unit k; the number of the unit at the bottom is 1 and increases by one along the axis; and the top unit displacement is the surface displacement when k=n.

From the above, it can be seen that the mensuration consists of multi-points of fixed measurement and that the measuring device does not have a linear motion. The position of the pipe axis is directly calculated by the pre-set measurement unit spacing L, and no additional measurement is required.

## 3. Design of the Measuring Instrument

### 3.1. Overview of the Instrument

**Figure 3:**
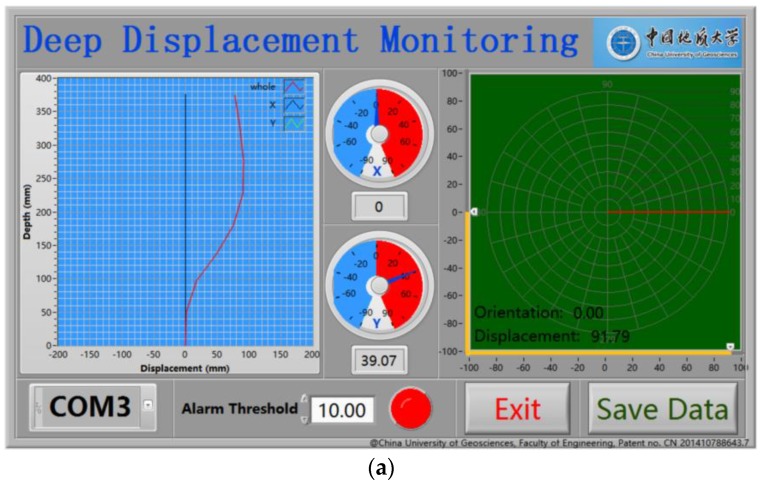

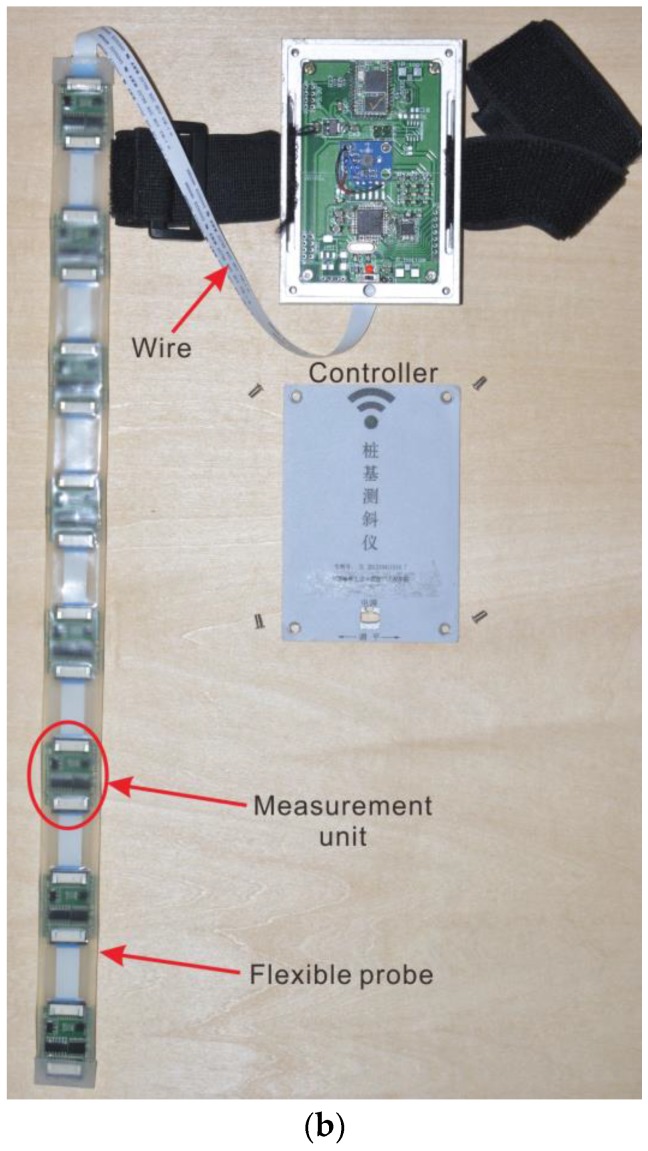
Overview of the instrument: (**a**) PC software; (**b**) flexible inclinometer.

As shown in Figure 3, the whole measurement device includes a flexible probe, controller, and PC software. The flexible probe contains several gravitational acceleration measurement units that are connected by an RS-485 communication bus. The controller is an microcontroller unit (MCU) circuit that contains the functions of 485 host and wireless communications. The MCU is the communication bridge between the probe and PC software, receiving command code from the PC software and writing it into measurement units or reading data from the measurement units and sending it to the software at the PC, and it is also a power supply for the probe. The function of the PC software is to calculate and draw the shape curve of the flexible probe. The flexible probe is the key part of the instrument.

Based on the measuring principle described above, the basic measurement unit of the instrument is a gravitational accelerometer. In the unit, a micro-electro-mechanical system (MEMS) acceleration sensor chip, MMA8451, is used to detect the 3 axis components of gravity. The unit is controlled by an MCU and connected to the 485 bus.

### 3.2. Gravitational Acceleration Measurement Unit

The output of the MMA4851 is an inter-integrated circuit (IIC) digital signal that can be read directly by the MCU. As fast as 400 kHz, the highest sample frequency of the MMA4851 is far beyond the landslide survey sampling frequency; therefore, the weighted average of a large amount of sampling data can be used to improve the accuracy as in equation

(8) $$data_{n}=data_{n-1}\cdot \left(1-p\right)+data_{c}\cdot p,$$

where $data_{n}$ is weighted average data in the *n*th sampling, $data_{c}$ is current sampling data, and p is the weight of the current sampling data. As seen from Equation (8), the smaller p is, the more insensitive to transient signals, but it will also spend more time reaching a stable value. Fortunately, inclination in a landslide can be regarded as a quasi-static measurement. Every jump signal is a singularity and should be smoothed out to allow more time to obtain more accurate measurement results. The suggested value cut-off for p is p < 0.05. According to the actual test, when p = 0.01, the delay time for reaching a stable value is approximately 3 seconds and the accuracy of the angle is 0.02 degrees, which is well within the measurement requirements of the landslide model test.

### 3.3. Composition and Encapsulation of the Probe

Depending on the specific application conditions, the length of the probe and the interval of the units can be customized before encapsulation. Generally, the length of the probe is slightly longer than the thickness of the landslide body. Shorter is better for the interval of units, but shorter intervals will require many more units. The design interval here for the model test is 50 mm, while 500 mm is used for the field monitoring.

**Figure 4:**
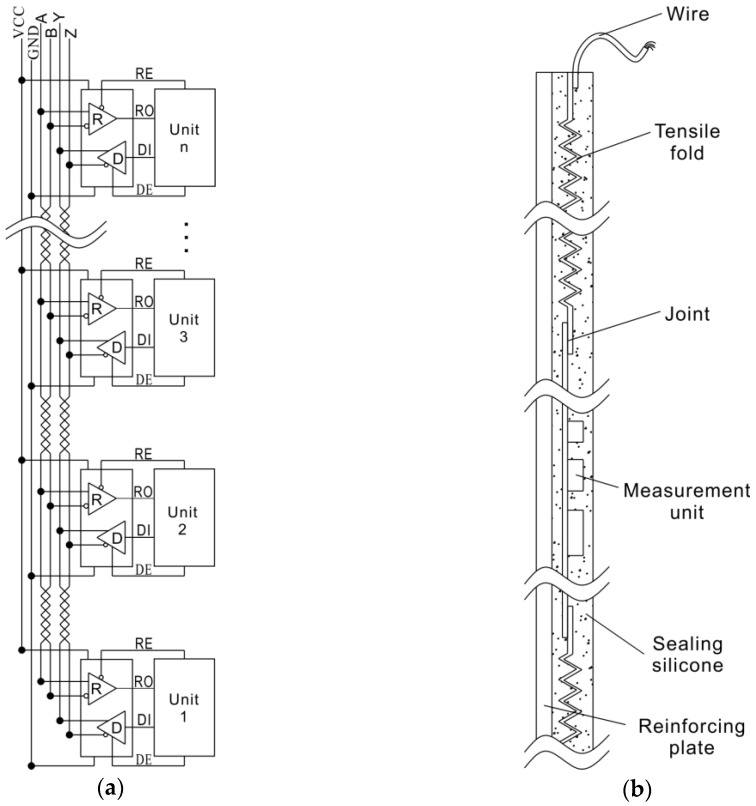
Composition and encapsulation diagrams of the probe: (**a**) Electrical connection diagram of the probe; (**b**) encapsulation structure diagram of the probe.

The encapsulation structure of the probe is shown in Figure 4b. The whole electrical assembly is put into a “U” shaped silicone tube and potted with liquid silicone that soon solidifies. The encapsulated probe can be seen in Figure 3b. Silicone gives the probe good elasticity, a high deformation ability, and good waterproof performance. However, the silicone can cause trouble in installation, such as stretching and twisting, especially when the probe is long. To address this problem, an auxiliary substrate made from aluminum or steel is used to reinforce a long probe.

## 4. Verification and Correction of Measurement Results

At present, the mature calibration instrument can only carry out angle verification for a single measuring unit, and it cannot calibrate the final displacement measurement for the whole probe. Therefore, a method based on the checkered paper tracer is proposed to verify the measurement accuracy of the probe.

### 4.1. Method for Verification of Measurement Results

**Figure 5:**
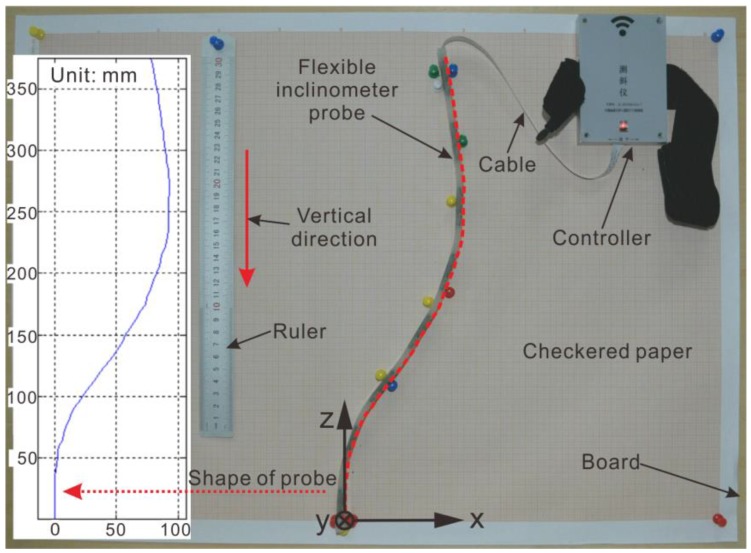
Verification method of measurement results for the flexible inclinometer probe.

It is indispensable to calibrate each measuring unit with a conventional angle calibration before the probe is packaged, but such measurements cannot fully guarantee the overall measurement accuracy of the probe after packaging. The calibration method for the whole probe is shown in Figure 5. This calibration involves placing the checkered paper on the vertical plank, adjusting the paper until its vertical lines are perpendicular to the horizontal plane, bending the probe according to the shape to simulate the landslide displacement state, and fixing the shape of the probe with tacks on the checkered paper. The amount of displacement determined by the fixed probe can be read through the checkered paper directly.

The shape of the probe in Figure 5 is shown by the red dotted line. With the bottom at zero, the displacement data identified by the grid paper are read at 5 mm intervals and are listed in Table 1. Meanwhile, the measurement data in the same state can be saved with computer software. The relative error of the measurement data is evaluated by using the data listed in Table 1 as the reference data $D_{b}$.

(9) $$\delta =\frac{D-D_{b}}{D_{b}}\cdot 100\%,$$

where D is the displacement curve measured by the probe. The relative error curve δ reflects the accuracy of the measurement results, and the curve δ through several checksums can be directly used to compensate for the measurement results to correct the drawing data of the PC software (Figure 3a).

In addition, it is necessary to evaluate the accuracy of the measurement results through the variance of the measured data relative to the reference data, which is

(10) $$S=\frac{1}{N}\sum_{i=1}^{N}{\left(D_{i}-D_{b,i}\right)}^{2},$$

where S is variance, N is the length of the measured data column, and $D_{b,i}$ is the reference value for the depth *i* position. The variance S can only be used to reflect the accuracy of the measurement results, and it cannot be used to correct the measurement results.

### 4.2. Correction of Measurement Results

**Figure 6:**
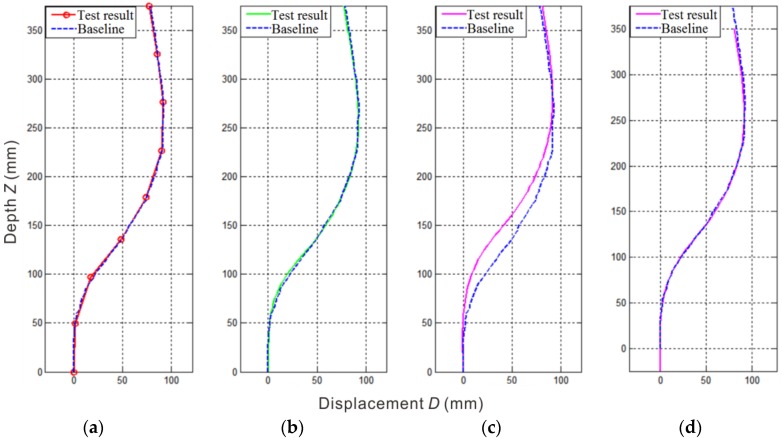
Displacement curves obtained with different calculation methods: (**a**) original displacement polyline; (**b**) displacement curve calculated by Equation (11); (**c**) displacement curve calculated by Equation (12); (**d**) displacement curve calculated by Equation (13).

Table 2 shows the data measured with the flexible inclinometer probe in the state shown in Figure 5 corresponding to the displacement curve shown in Figure 6a. Limited by the number of measurement points, the displacement curve is actually a polyline based on the interval length between the measuring points, which will affect the accuracy of the measurement results, especially in the case where the interval distance is large. To obtain a smoother displacement curve, the cubic spline interpolation method is used to interpolate the measurement results. Due to its good stability and convergence, cubic spline interpolation is well suited for correcting the measurement results.

According to the measuring principle, the data obtained by the inclinometer probe are the inclination angles of the measurement units, while the displacement curve is the result of the angle projection. Therefore, there are two cases of interpolation: one is to first calculate and then interpolate for the displacement curve, and the other is to first interpolate for the angular sequence and then calculate the displacement curve. These two calculation methods are respectively shown in Equations (11) and (12).

(11) $$\left\{ \begin{array}{l} ①:D_{0,i}=\sum_{i=1}^{k}L_{i}\cdot \sin\left(\alpha_{i}\right)\\ ②:Z_{0,i}=\sum_{i=1}^{k}L_{i}\cdot \cos\left(\alpha_{i}\right)\\ ③:D=spline\left(Z_{0},D_{0},\left[0:\Delta z:\max\left(Z_{0}\right)\right]\right) \end{array}\right.,\left(k=1,2,3,\ldots ,N\right).$$

In Equation (11), the displacement curve is calculated from the measuring angle sequences by steps ① and ②. Step ③ interpolates to the displacement curve ($D_{0}$, $Z_{0}$), and re-sampling is performed at an equal-spaced depth Δz. To align with the baseline data, Δz here should be consistent with the sampling interval of the baseline data, which means Δz = 5 mm. The displacement curve calculated from Equation (11) is shown in Figure 6b. Compared with the displacement curve shown in Figure 6a, the displacement curve calculated by Equation (11) is smoother, eliminating some parts of the error. However, the curve is still controlled by the control points on the polyline in Figure 6a. Thus, the error caused by the control points cannot be eliminated.

(12) $$\left\{ \begin{array}{l} ①:\alpha_{I}=spline\left(\left[0:1:N],\alpha ,[0:\frac{1}{m}:N\right]\right)\\ ②:D_{0,i}=\sum_{i=1}^{k}\frac{L}{m}\cdot \sin\left(\alpha_{I,i}\right)\\ ③:Z_{0,i}=\sum_{i=1}^{k}\frac{L}{m}\cdot \cos\left(\alpha_{I,i}\right)\\ ④:D=spline\left(Z_{0},D_{0},\left[0:\Delta z:\max\left(Z_{0}\right)\right]\right) \end{array}\right.,\left(k=1,2,3,\ldots ,m\cdot N\right).$$

In Equation (12), m is an interpolation of multiples of angle sequences and $\alpha_{I}$ is the interpolated angle sequence. The angle sequence is interpolated first in step ①; then, the displacement curve is calculated through the interpolated angle sequence in steps ② and ③. The displacement curve is resampled according to the cubic spline interpolation method in step ④. The purpose of resampling is to align the calculation results with the reference data; however, no substantial interpolation of the displacement curve is made in step ④. Through the displacement curve calculated by Equation (12) shown in Figure 6c, it is clear that the calculated displacement curve has a certain phase difference with the reference curve at the depth z.

The angle sequence interpolation is based on the continuity of the probe angle change (first-order derivative of the angle curve), which means that there is no abrupt change in the angle between adjacent measurement units, and it forms a smooth transition. In Equation (11), the angle distribution of the entire unit body length L is replaced by the angle of the measuring point so that there is no phase difference. When the angle sequence is interpolated by Equation (12), the interval L between the measurement points is controlled by the angle of the two measurement units at the same time; therefore, it can be concluded that the phase difference is L/2. The displacement curve represented by Equation (12) is shifted downward by L/2 to obtain equation:

(13) $$\left\{ \begin{array}{l} ①:\alpha_{I}=spline\left(\left[0:1:N],\alpha ,[0:\frac{1}{m}:N\right]\right)\\ ②:D_{0,i}=\sum_{i=1}^{k}\frac{L}{m}\cdot \sin\left(\alpha_{I,i}\right)\\ ③:Z_{0,i}=\sum_{i=1}^{k}\frac{L}{m}\cdot \cos\left(\alpha_{I,i}\right)-\frac{L}{2}\\ ④:D=spline\left(Z_{0},D_{0},\left[0:\Delta z:\max\left(Z_{0}\right)\right]\right) \end{array}\right.,\left(k=1,2,3,\ldots ,m\cdot N\right),$$

and the corresponding displacement curve is shown in Figure 6d.

As seen from Figure 6b,d, the accuracy of the measurement is improved by these two correction algorithms. To accurately evaluate the accuracy of the two algorithms, the mean variance of the two types of calculation results with respect to the reference data is calculated using Equation (10). The mean variance results, 1.42 mm for Figure 6b and 1.17 mm for Figure 6d, show that Equation (13) is more accurate than Equation (11).

## 5. Application of the Flexible Inclinometer Probe

### 5.1. Arrangement Methods of the Flexible Probe in the Physical Model of a Landslide

**Figure 7:**
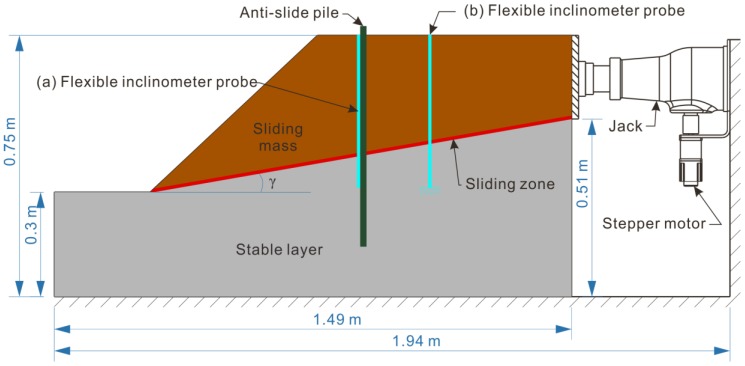
Arrangement of the flexible probe in the physical model test of a landslide. (**a**) Flexible inclinometer probe in pile-containing structural model; (**b**) Flexible inclinometer probe in non-pile structural model.

The flexible probe can be arranged in pile-containing structural model (Figure 7 probe a) and non-pile structural model (Figure 7 probe b). The pile-containing structural model focuses on monitoring the deformation and displacement of the pile body, and the non-pile structural model focuses on monitoring the deformation and displacement of the landslide body itself.

In the pile-containing structural model, the flexible probe is directly attached to the pile body for monitoring, as shown in Figure 7 (probe a). The bottom of the probe is the reference point for the displacement calculation, and its position must be fixed during the test. Therefore, the arrangement of the bottom of the probe is at least imbedded into the section where the deformation of the pile is negligible.

In the non-pile structural model, the probe is directly embedded in the landslide body, as shown in Figure 7 (probe b). The probe has the best coupling for stress and deformation when its wide surface is aligned with the main slip direction of the landslide. The bottom of the probe should be embedded in the bedrock to ensure that it does not move during the test. When the probe is buried, there is no strict requirement for verticality, but to take full advantage of the accelerometer’s most effective sensitivity range, it is strongly recommended that the initial inclination angle of the probe is less than 30°.

### 5.2. Description of the Physical Model Test Situation

Unfortunately, guided by the specific project needs, only the probe arranged in the pile-containing structural model has been tested so far. The physical model, containing a sliding mass, a sliding zone and a stable layer, has a size of 1.49 m long, 0.75 m height and 0.5 m width. The sliding mass is mixed by coarse sand and clay with a ratio of 1:1, having the properties of approximate 9 kPa for its cohesion and 29° for its friction angle. The stable layer is made of concrete, composed by coarse sand, cement, gesso, and water with a ratio of 10:1:2:3.

### 5.3. Testing Process and Test Results

**Figure 8:**
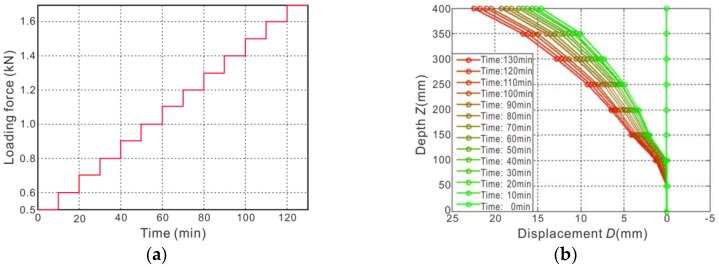
The loading process and displacement monitoring results of the model test: (**a**) test loading process; (**b**) displacement curves.

The loading process and the displacement monitoring results of a physical model test are shown in Figure 8. After the model was built, the initial state of the flexible inclinometer probe was read as a displacement zero before loading, as shown in the “Time: 0 min” curve in Figure 8b. During the test, the model was graded and loaded according to the curve shown in Figure 8a. The initial stress of the loading was set at 0.5 kN and then increased by 0.1 kN. Each stage of the loading time lasts 10 min, and the displacement is measured once by the flexible probe before the start of the next stage of loading. The whole loading process continues for 130 min, and the monitored displacement curves are shown in Figure 8b.

**Figure 9:**
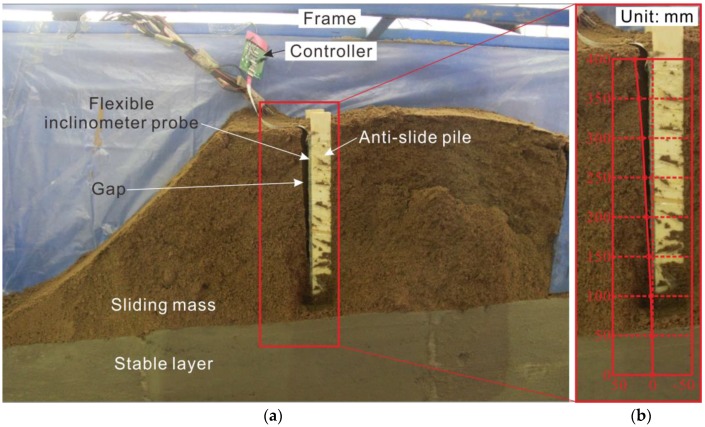
Physical model profile after unloading at the end of the test and comparison: (**a**) model longitudinal pic; (**b**) measurement curve at 130th min vs. the gap.

After the test, by opening the model frame side baffle and cutting out the side of the soil, the profile is revealed, as shown in Figure 9. Since the pile mainly produces elastic deformation and the soil mainly produces plastic deformation, the pile is restored to the original state when the loading force is unloaded, while the soil body retains the maximum deformation state, leaving a gap that can be measured directly. The shape of the gap shows the deformation and distribution of the pile, corresponding to the measurement for “time: 130 min” in the red curve in Figure 8b.

As shown in Figure 9b, the displacement curve measured by the flexible probe is highly coincident with the gap. The maximum top displacement is 22.44 mm, which is in close agreement with the corresponding measurement of the gap width of 22.5 mm (measured by the vernier caliper). Therefore, the feasibility and accuracy of the flexible inclinometer probe used for displacement monitoring in a landslide physical model test are proven.

## 6. Discussion

Limited by the basic principle, the flexible inclinometer probe cannot measure the displacement on the z-axis or the rotation around the z-axis, which should be considered in its application. If z-axis displacement is necessary, additional instruments are required as a supplement. Concerning the rotation, self-twisting of the flexible inclinometer probe should be avoided during installation, and the initial orientation of the probe should be determined, recorded, and used as a reference direction for the measurement. In addition, encapsulated as a flat belt, the x-axis of the probe has the best deformation characteristics; thus, although the probe can measure the displacements on both the x and y axes, it is strongly recommended to align the x-axis with the main slip direction as much as possible to obtain the best deformation coupling between the probe and the sliding body.

In the physical model test, the x-axis just coincides with the main slip direction of the landslide, so it is reduced to 2-D displacement measurement, which is also consistent with most physical model test environments. However, this is not to say that the flexible inclinometer probe is only suitable for measurements with a single direction [27,31]. According to the principle of conversion, the combined displacement can be converted from both x-angle and y-angle by Equation (6).

Additionally, both the flexible inclinometer probe and the data interpolation method can be applied to landslide field monitoring. A dedicated reinforced structure for the field installation is being designed and constructed, and it will be installed in an actual landslide soon. The spline interpolation method and its interpolation order are fully applicable to on-site measurement. Thanks to the simplicity of its silicone encapsulation structure, the flexible inclinometer probe will be more economical than traditional inclinometers for landslide field monitoring.

## 7. Conclusions

Flexible inclinometer probe development is driven by the requirements of deep displacement measurements in the physical model test of a landslide. With the advantages of good deformation coupling, large deformation capacity, and automatic measurement, the flexible probe is a good match for the physical model test. The key features are as follows.

(1) Analysis of the measuring principle. With stability in time and space, gravitational acceleration is the ideal reference physical quantity for attitude measurement. The flexible probe senses inclination information based on the gravity component and can accurately reflect the relationship between the measurement data and deep displacement, including size and direction. The number and intervals of the measurement units can be flexibly customized to match the different operating conditions.
(2) Design of the flexible inclinometer probe. The key sensor is a MEMS chip with digital output and a high sampling rate; thus, a large amount of data can be used to calculate the weighted average to confirm the accuracy with an acceptable time delay. Encapsulated by silicone, the flexural rigidity of the probe can be ignored relative to the landslide body, with displacement coupling well to the landside body.
(3) Calibration and correction of the measurement result. A visual calibration method was proposed by comparing the shape curve read from checkered paper. There are two ways to correct the measurement results, and the optimal correction method is to interpolate the angle data by the cubic spline and then calculate the displacement curve and move up L/2.
(4) Application of the flexible inclinometer probe. The flexible probe can be buried in a landslide physical model directly or attached to an anti-sliding pile. The test conducted by attaching the probe to the pile verifies that the flexible probe is a good match with the landside model, and the accuracy is lower than 1 mm/400 mm. With these functions and features, the flexible probe designed in this paper is not only suitable for a landslide model test but also for field monitoring.

## Figures and Tables

**Table 1:** Baseline data read by checkered paper.

Depth (mm)	Db,x * (mm)	Depth (mm)	Db,x (mm)	Depth (mm)	Db,x (mm)	Depth (mm)	Db,x (mm)	Depth (mm)	Db,x (mm)
0	0.0	80	11.0	160	64.0	240	92.0	320	87.0
5	0.0	85	12.8	165	67.0	245	92.0	325	86.8
10	0.0	90	15.5	170	70.0	250	92.0	330	85.8
15	0.0	95	19.0	175	73.5	255	92.0	335	85.0
20	0.0	100	22.5	180	75.0	260	92.0	340	84.4
25	0.0	105	25.5	185	77.0	265	93.0	345	84.0
30	0.0	110	30.0	190	79.0	270	93.0	350	83.0
35	0.2	115	34.0	195	80.5	275	93.0	355	82.0
40	0.7	120	36.5	200	83.0	280	92.0	360	81.0
45	1.4	125	41.1	205	85.0	285	92.0	365	80.5
50	2.2	130	45.0	210	86.0	290	91.0	370	79.5
55	2.2	135	49.0	215	87.0	295	90.8	375	78.0
60	3.5	140	52.0	220	89.0	300	90.0		
65	6.0	145	55.0	225	90.5	305	89.2		
70	7.0	150	57.0	230	91.5	310	88.1		
75	8.9	155	61.0	235	92.0	315	88.0		

* $D_{b,x}$ is the x-axis displacement data of baseline.

**Table 2:** Measurement results data.

Probe Position (mm)	$\alpha_{x}$ (rad)	$D_{x}$ (mm)	$D_{y}$ (mm)	Z (mm)
0	0.0000	0.00	0	0.00
50	0.0330	1.65	0	49.97
100	0.3237	17.56	0	97.38
150	0.6819	49.07	0	136.19
200	0.5405	74.80	0	179.07
250	0.3126	90.18	0	226.64
300	0.0323	91.79	0	276.62
350	-0.1256	85.52	0	326.22
400	-0.1554	77.78	0	375.62
